# Comparison of prognostic impact of anatomic location of the pancreas on postoperative pancreatic fistula in laparoscopic and open gastrectomy

**DOI:** 10.1186/s12876-020-01476-9

**Published:** 2020-10-06

**Authors:** Jun Kinoshita, Takahisa Yamaguchi, Hiroto Saito, Hideki Moriyama, Mari Shimada, Shiro Terai, Koichi Okamoto, Shinichi Nakanuma, Isamu Makino, Keishi Nakamura, Hidehiro Tajima, Itasu Ninomiya, Sachio Fushida

**Affiliations:** Department of Gastroenterological Surgery, Division of Cancer Medicine, Graduate School of Medical Science, Kanazawa University, 13-1 Takara-machi, Kanazawa, Ishikawa 920-8641 Japan

**Keywords:** Postoperative pancreatic fistula (POPF), Laparoscopic gastrectomy (LG), Open gastrectomy (OG), Amylase level in the drained fluid (D-Amy)

## Abstract

**Background:**

Postoperative pancreatic fistula (POPF) is a serious complication after gastric cancer surgery. The current study aimed to investigate the significance of the anatomic location of the pancreas as a predictor for POPF in both laparoscopic gastrectomy (LG) and open gastrectomy (OG).

**Methods:**

In total, 233 patients with gastric cancer were assessed retrospectively. We measured the maximum vertical (P-L height; PLH) and horizontal length (P-L depth; PLD) between the upper border of pancreas and the root of left gastric artery on a preoperative CT in the sagittal direction. The maximum length of the vertical line between the surface of the pancreas and the aorta (P-A length), previously reported as prognostic factor of POPF, was also measured. We investigated the correlations between these parameters and the incidence of POPF in LG and OG groups.

**Results:**

Among the patients in this study, 118 underwent OG and 115 underwent LG. In LG, the median PLH and P-A length in patients with POPF were significantly longer compared with those without POPF (*p* = 0.026, 0.034, respectively), but not in OG. There was no significant difference in the median PLD between the patients with or without POPF in both LG and OG. The multivariate analysis demonstrated that PLH (odds ratio [OR] 4.19, 95% confidence interval [CI] 1.57–11.3, *P* = 0.004) and P-A length (OR 4.06, 95%CI 1.05–15.7, *P* = 0.042] were independent factors for predicting POPF in LG. However, intraoperative blood loss (OR 2.55, 95%CI 1.05–6.18, *P* = 0.038) was extracted as an independent factor in OG. The median amylase level in the drained fluid (D-Amy) were significantly higher in patients with high PLH(≥12.4 mm) or high P-A length (≥45 mm) compared with those with low PLH or low P-A length in LG. However, there were no differences in the D-Amy levels by PLH or P-A length in OG patients.

**Conclusions:**

The anatomic location of the pancreas is a specific and independent predictor of POPF in LG but not in OG. PLH is a simple parameter that can evaluate the anatomic position of the pancreas, and it may be useful for preventing POPF after LG.

## Background

Postoperative pancreatic fistula (POPF) is a complication that occurs fairly frequently in gastrectomy with radical lymphadenectomy, and it can lead to serious complications such as bleeding, intra-abdominal abscesses, and anastomotic leakage. Body mass index (BMI), extension of lymphadenectomy, tumor location, and stage are considered to be conventional risk factors for POPF after gastrectomy [1–6]. Several articles have reported a close relationship between pancreatic fistula and the amylase level in the drained fluid (D-Amy), suggesting that postoperative D-Amy levels may be used as an indicator of pancreatic injury after gastrectomy [3, 6–10].

There have been several reports on the incidence of POPF in laparoscopic gastrectomy (LG) and open gastrectomy (OG) for gastric cancer. Based on the JCOG 0703 trial, which was a Japanese phase II trial that was performed to investigate the safety and feasibility of LG for early gastric cancer, the POPF frequency after LG was as low as 1.1%, and this was considered to be acceptable compared with the POPF frequency after OG [11]. The incidence of grade 3–4 POPF (CTCAE v 4.0) after LG was reported to be 0.4%, which was equivalent to OG in for short-term outcomes from a phase III randomized controlled trial that was performed to investigate the efficacy of LG compared with OG for early gastric cancer (JCOG 0912) [12]. However, it is unclear whether the results of these clinical trials that were conducted by a high-volume center and experienced surgeons are applicable to the surgical community in general. Several studies have reported that POPF occurs more frequently after LG compared with after OG [7, 13–15]. Additionally, a prospective cohort study comparing LG and OG using National Clinical Database (NCD) in Japan reported that the incidence of POPF after LG was 2.2%, which was significantly more common compared with after OG (1.0%) in the real world [16].

Potential risks using the laparoscopic approach include reduced tactile sensation, a limited operative view, and limitations of the instrument’s axis. When performing suprapancreatic lymph node dissection in particular, traction or compression of the pancreas is sometimes required to show a good operative field in the suprapancreatic area, and excessive compression or mobilization can cause blunt damage to the pancreas, resulting in POPF. It has been reported that the anatomical position of the pancreas and the celiac or common hepatic artery on computed tomography (CT) images are involved in the development of POPF after LG, and these factors were identified as independent predictors of POPF [17, 18]. Kumagai et al. measured the distance between the aorta and pancreas (P-A length) on CT sagittal section images, and they reported that the P-A length was significantly correlated with POPF [18].

Based on these data, we hypothesized that the incidence of POPF in LG may be more strongly affected by the anatomic location of the pancreas compared with OG. However, because previous reports only involved LG patients, the relationship between the anatomic location and the incidence of POPF has not yet been determined in OG.

The current study aimed to investigate the significance of the pancreas location as a predictor of POPF in both LG and OG and to determine whether the anatomic location of the pancreas is a specific risk factor for POPF in LG. We evaluated the anatomic location of the pancreas in relation to the root of the left gastric artery on a CT image with a sagittal view, and investigated its impact on the incidence of POPF and D-Amy after LG compared with OG.

## Methods

### Ethics

This study conformed to the ethical guidelines of the World Medical Association Declaration of Helsinki—Ethical Principles for Medical Research Involving Human Subjects. Written informed consent for the surgery and the use of clinical data, as required by the Review Board of Kanazawa University (Kanazawa, Japan), was obtained from all patients.

### Patients

A single-center retrospective cohort study was performed between April 2015 and December 2019. During this study period, 321 patients underwent gastrectomy for histologically proven gastric cancer at the Department of Gastroenterological Surgery, Kanazawa University. Because this study was intended to evaluate the incidence of POPF after standard gastrectomy and the anatomic location of the pancreas, we excluded patients with remnant gastric cancer, patients who underwent limited lymphadenectomy because of insufficient physical condition or palliative surgery, patients who underwent para-aortic lymph node dissection, and patients who underwent combined resection resulting from the invasion of other adjacent organs, except for cholecystectomy and splenectomy. Ultimately, 233 patients who underwent radical gastrectomy for gastric cancer were enrolled. Among them, 118 patients underwent OG and 115 patients underwent LG. Patient data were retrieved retrospectively from our database and the patient’s hospital records. The present study was approved by the Institutional Review Board of Kanazawa University.

### Surgical procedures

Clinical stages of the patients were determined in accordance with the Japanese Classification of Gastric Cancer, 3rd English edition [19] and the UICC TNM classification, 8th edition. The type of gastrectomy and the extent of lymph node dissection were determined in accordance with the Japanese Gastric Cancer Treatment Guidelines 2018 (ver. 5) [20]. LG was performed using D1 plus lymph node dissection for patients with clinically diagnosed T1N0M0 cancer or using D2 lymph node dissection for those with T2N0M0 or T1N1M0 cancer. OG was used for patients with stage IB or higher. At the end of the operation, the abdominal cavity was washed with warm saline, as follows: 1 L for patients who underwent LG and 5 L for those who underwent OG. A closed drain was routinely placed in the peripancreatic space to allow drainage of excess fluid. During the study period, the drain fluid was analyzed to determine the D-Amy levels on postoperative day (POD) 1 and POD 3.

### Evaluation of the pancreatic anatomical parameters

**Fig. 1:**
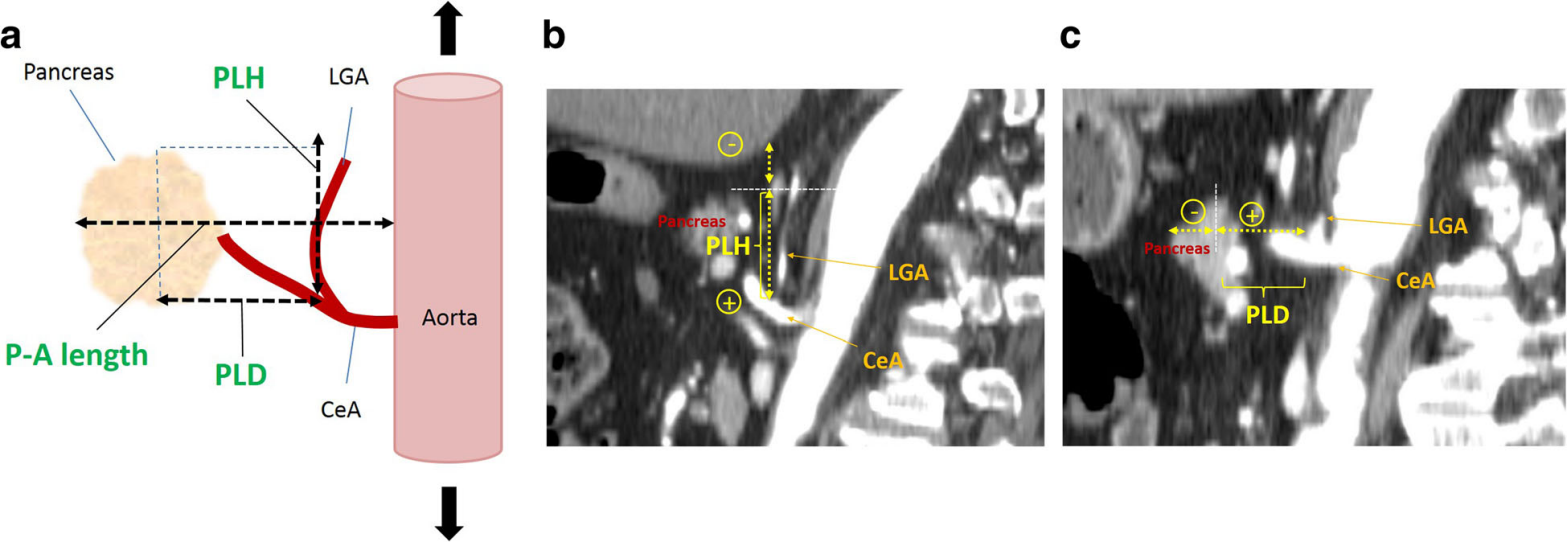
Evaluation of pancreatic anatomical parameters on CT images in the sagittal direction. **a** The maximum vertical and horizontal lengths between the upper border of pancreas and the root of left gastric artery were measured as P–L height (PLH) and P-L depth (PLD). The maximum length of the vertical line between the surface of the pancreas and the aorta (P-A length). **b** Representative images of PLH. The caudal direction was recorded as a positive number and the cranial direction as a negative number starting from the upper border of pancreas. **c** Representative images of PLD. The dorsal direction was recorded as a positive number and the ventral direction as a negative number starting from the upper border of the pancreas

We reviewed the preoperative contrast-enhanced abdominal CT images in the sagittal view for all of the patients who were included in the current study. We measured the maximum vertical length between the upper border of the pancreas and the root of the left gastric artery (P–L height: PLH), which represents the height of the step looking down toward the root of the left gastric artery over the pancreas. We also measured the maximum horizontal length between the upper border of pancreas and the root of left gastric artery (P–L depth: PLD), which represents the depth of the root of the left gastric artery over the pancreas. Representative images of these parameters are shown in Fig. 1a-c. For PLH, the caudal direction was recorded as a positive number and the cranial direction was recorded as a negative number starting from the upper border of the pancreas. Similarly, PLD was recorded as a positive number in the dorsal direction and a negative number in the ventral direction starting from the upper border of pancreas. We also measured the maximum length of the vertical line between the pancreatic body surface and the aorta (P–A length), as previously reported by Kumagai et al. [18].

### Definition of POPF

POPF was defined as peripancreatic fluid collection with inflammatory change that was detected using a CT scan without clinical or radiographic evidence of anastomotic leakage. Although we defined POPF based on imaging studies and the clinical course, the criteria for POPF based on the International Study Group of Postoperative Pancreatic Fistula (ISGPF) were also applied in the current study because this is a well-known classification scheme among the surgical community [21–23]. Briefly, POPF was diagnosed when the patients fulfilled the following criteria: a D-Amy level on POD 3 of more than three times the upper normal serum value (normal serum value: 40–113 U/L). The severity of POPF was graded, in accordance with the ISGPF definition, as follows: BL (“biochemical leak”; no clinical impact); grade B (requires a change in management or adjustment of the clinical pathway); and grade C (requires a major change in clinical management or deviation from the normal clinical pathway) [24].

### Statistical analysis

The Mann–Whitney U-test and χ2 test were employed to evaluate differences in continuous and categorical variables, respectively. To evaluate the sensitivity and specificity of the PLH for detecting the development of POPF, receiver operating characteristics (ROC) curves were calculated, and the Youden index was estimated to determine the optimal cut-off values [15]. All variables with a *p* value of < 0.1 in the univariate analysis were entered into a multivariate analysis. The multivariate analysis used a logistic regression model to investigate the factors that were associated with the incidence of POPF. Additionally, *p* < 0.05 was considered to indicate statistical significance. The statistical analyses were performed using the SPSS software program (version 19.0; SPSS, Chicago, IL, USA).

## Results

### Patient characteristics and postoperative pancreatic fistula

There were 233 patients enrolled into the study, and the details of their clinical and pathological features are shown in Table 1. When the patient background of the OG and LG groups were compared, there were no differences in sex, body mass index (BMI), ASA-physical status, and comorbidity, whereas a significant difference was observed for age (OG, 71 [39–90] years vs. LG, 67 [42–88] years; *p* < 0.001), clinical stage (I/II/III/IV: OG, 28/35/51/4 vs. LG, 115/0/0/0; *p* < 0.001), surgical procedure (DG/PPG/PG/TG: OG, 60/0/20/38 vs. LG, 77/3/25/10; *p* < 0.001), extent of lymphadenectomy (D1+/D2: OG, 38/80 vs. LG, 112/3; *p* < 0.001), operative duration (OG, 224 min [97–433] vs. LG, 277 min [159–432]; *p* < 0.001), blood loss (OG, 200 [0–1370] vs. LG, 5 [5–380], *p* < 0.001), and perioperative transfusion (Yes/No: OG, 12/106 vs. LG, 1/114; *p* = 0.002). The OG group included more patients with an older age, advanced cancer, total gastrectomy, or D2 dissection, which are considered to be conventional risk factors for POPF, compared with the LG group. For LG patients, the D-Amy levels on POD 3 were significantly higher compared with OG (LG 134 [24–5670] IU/L vs. OG 123 [12–2855] IU/L, *p* = 0.016). However, POPF occurred in 27 OG patients (22.9%), including BL (*n* = 16), grade B (*n* = 10), and grade C (n = 1), and in 31 LG patients (27.0%), including BL (*n* = 24) and grade B (*n* = 7), and there was no significant difference in the incidence of POPF between OG and LG. There was also no significant difference in the pancreatic anatomical parameters PLH, PLD, and PA-length between OG and LG.

**Table 1:** Patient characteristics

	Open gastrectomy (***n*** = 118)	Laparoscopic gastrectomy (***n*** = 115)	*p*
**Age** (years) median (range)	71 (39–90)	67 (42–88)	< 0.001
**Sex** (male/female)	76/42	81/34	0.326
**ASA-PS** 1/2/3	9/91/18	8/99/8	0.122
**Comorbidity**			
Diabetes mellitus	18 (15.3%)	16 (13.9%)	0.641
Cardiovascular disease	12 (10.2%)	15 (13.0%)	0.493
Respiratory disease	18 (15.2%)	9 (7.8%)	0.077
Renal dysfunction	3 (2.5%)	1 (0.8%)	0.322
Total	51 (43.2%)	41 (35.7%)	0.237
**Body mass index (kg/m**^**2**^**)**			
Median (range)	22.3 (17.2–34.7)	23.2 (16.4–31.5)	0.484
**Tumor location**			
Upper third (%)	33 (28.0%)	27 (23.5%)	
Middle third (%)	45 (38.1%)	32 (27.8%)	0.066
Lower third (%)	40 (33.9%)	56 (48.7%)	
**cStage**			
I/II/III/IV	28/35/51/4	115/0/0/0	< 0.001
**Surgical procedure**			
DG (%)	60 (50.8%)	77 (67.0%)	
PPG (%)	0 (0%)	3 (2.6%)	
PG (%)	20 (16.9%)	25 (21.7%)	< 0.001
TG (%)	38 (32.1%)	10 (8.7%)	
**Lymphadenectomy**			
D1+/D2	38/80	112/3	< 0.001
**Operative duration** (min)			
Median (range)	224 (97–433)	277 (159–432)	< 0.001
**Blood loss** (g)			
Median (range)	200 (0–1370)	5 (5–380)	< 0.001
**Perioperative transfusion**			
Yes/No	12/106	1/114	0.002
**D-Amy** (IU/L)			
POD1 Median (range)	320 (43–16,764)	419 (38–12,154)	0.234
POD3 Median (range)	123 (12–2855)	134 (24–5670)	0.016
**POPF**			
Total	27 (22.9%)	31 (27.0%)	
BL	16 (13.6%)	24 (20.9%)	
Grade B	10 (8.5%)	7 (6.1%)	0.239
Grade C	1 (0.8%)	0 (0%)	
**PLH** (mm) median (range)	8.3 (−26.7–34.9)	10.1 (−19.8–24.9)	0.615
**PLD** (mm) median (range)	11.4 (−5–25.8)	11.2 (0–27.1)	0.842
**P-A length** (mm) median (range)	35.5 (12.3–59.8)	35.8 (16.7–49.8)	0.716

*ASA-PS* ASA physical status, *DG* distal gastrectomy, *PPG* pylorus preserving gastrectomy, *PG* proximal gastrectomy, *TG* total gastrectomy, *D-Amy* amylase concentration in the drained fluid, *POD* postoperative day, *POPF* pancreatic fistula, *BL* biochemical leak, *PLH* the maximum vertical length between the upper border of pancreas and the root of left gastric artery, *PLD* the maximum horizontal length between the upper border of pancreas and the root of left gastric artery, *P-A* pancreas–aorta

### Anatomical parameter characteristics of the pancreas and POPF

**Fig. 2:**
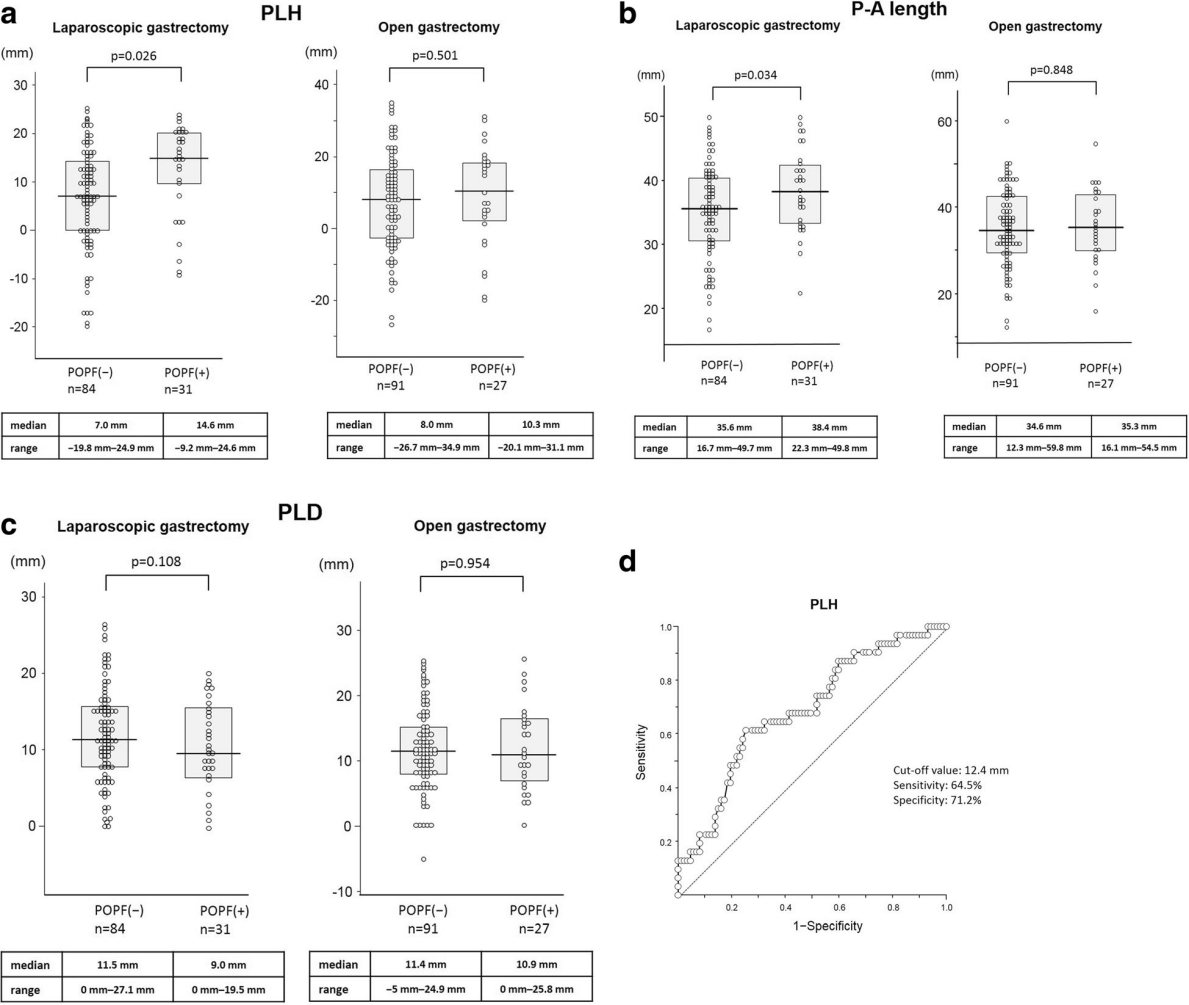
Characteristics of pancreatic anatomical parameters and POPF. **a** Summary of PLH values in patients with and without POPF after laparoscopic and open gastrectomy using a one-dimensional scatter plot and a comparison using the Mann–Whitney *U* test. **b** Summary of P-A length values in patients with and without POPF laparoscopic and open gastrectomy. **c** Summary of PLD values in patients with and without POPF laparoscopic and open gastrectomy. **d** Receiver operating characteristics curves of the PLH to predict POPF after laparoscopic gastrectomy. PLH, the maximum vertical length between the upper border of pancreas and the root of left gastric artery; P-A, pancreas-aorta; PLD, the maximum horizontal length between the upper border of pancreas and the root of left gastric artery; POPF, pancreatic fistula

Figure 2 shows the anatomical characteristics of the pancreas and POPF. The median PLH and P-A length in patients with POPF were significantly greater compared with those without POPF in LG patients (*p* = 0.026, 0.034, respectively; Fig. 2a and b). However, there was no significant difference in the median PLH between the two groups in OG. There was no significant difference in the median PLD between the two groups in both LG and OG (Fig. 2c). Using the POPF incidence as an endpoint, the area under the curve for the PLH was 0.686 using the ROC curve. The optimal cut-off value for predicting POPF was 12.4 mm for PLH, based on the Youden index, with a sensitivity of 64.5% and a specificity of 71.2% (Fig. 2d). The cut-off value of P-A length was set to 45 mm, as reported by Kumagai et al. [18].

### Risk factors for POPF

The results of univariate analyses of clinical factors are summarized in Table 2. For blood loss and surgical duration, we used the medians as the cut-off levels. PLH (≥12.4 mm) and P-A length (≥45 mm) were significantly associated with POPF in patients who underwent LG. However, univariate analysis showed that blood loss (≥200 mL) was a significant adverse factor for POPF in OG patients.

**Table 2:** Univariate analysis of the associations between clinical factors and postoperative pancreatic fistula

Open gastrectomy (***n*** = 118)				Laparoscopic gastrectomy (***n*** = 115)			
**Variables**	**OR**	**95%CI**	***p***	**Variables**	**OR**	**95%CI**	***p***
**Age** (years)				**Age** (year)			
≥75	1.48	0.59–3.74	0.402	≥75	1	0.90–10.7	0.073
< 75	1			< 75	3.11		
**Sex**				**Sex**			
Male	1.33	0.50–3.55	0.565	Male	1.22	0.48–3.08	0.677
Female	1			Female	1		
**BMI** (kg/m^2^)				**BMI** (kg/m^2^)			
≥25	1.40	0.51–3.81	0.510	≥25	2.49	1.00–6.19	0.051
< 25	1			< 25	1		
**ASA-PS**				**ASA-PS**			
2,3	2.29	0.64–8.15	0.203	2,3	1.09	0.20–5.93	0.921
1	1			1	1		
**Comorbidity**				**Comorbidity**			
Absent	1	0.51–2.94	0.653	Absent	1	0.54–2.96	0.591
Present	1.22			Present	1.26		
**cStage**				**cStage**			
I, II	1	0.73–4.38	0.201	I, II	N.E.	N.E.	N.E.
III, IV	1.79			III, IV			
**Procedure**				**Procedure**			
TG	1.14	0.48–2.73	0.767	TG	2.00	0.38–10.6	0.415
DG/PPG/PG	1			DG/PPG/PG	1		
**Operative duration** (min)				**Operative duration** (min)			
≥224	2.29	0.92–5.71	0.074	≥277	1.91	0.81–4.51	0.140
< 224	1			< 277	1		
**Blood loss** (g)				**Blood loss** (g)			
≥200	2.55	1.05–6.18	0.038	≥5	1.38	0.60–3.18	0.442
< 200	1			< 5	1		
**D-number**				**D-number**			
D2	1.19	0.47–3.02	0.719	D2	2.25	0.23–22.1	0.486
D1+	1			D1+	1		
**PLH** (mm)				**PLH** (mm)			
≥12.4	1.63	0.68–3.87	0.266	≥12.4	3.93	1.61–9.57	0.003
< 12.4	1			< 12.4	1		
**P-A length** (mm)				**P-A length** (mm)			
≥45	1.01	0.25–3.96	0.995	≥45	3.59	1.16–11.0	0.026
< 45	1			< 45	1		

*BMI* body mass index; DG, distal gastrectomy, *PPG* pylorus preserving gastrectomy, *PG* proximal gastrectomy, *TG* total gastrectomy, *ASA-PS* ASA physical status, *PLH* the maximum vertical length between the upper border of pancreas and the root of left gastric artery, *P-A* pancreas–aorta

Factors with *P* < 0.1 were then selected as co-variables in a multivariate analysis, as shown in Table 3. The multivariate analysis demonstrated that PLH (odds ratio [OR] 4.19, 95% confidence interval [CI] 1.57–11.3, *p* = 0.004) and P-A length (OR 4.06, 95%CI 1.05–15.7, *p* = 0.042) were independent predictive factors for POPF in LG patients. Intraoperative blood loss (OR 2.55, 95%CI 1.05–6.18, *p* = 0.038) was extracted as an independent predictive factor in OG patients.

**Table 3:** Multivariate analysis of the associations between clinical factors and postoperative pancreatic fistula

Open gastrectomy (***n*** = 118)				Laparoscopic gastrectomy (***n*** = 115)			
Variables	OR	95%CI	*p*	Variables	OR	95%CI	*p*
**Operative duration** (min)				**Age** (year)			
≥224	1.92	0.75–4.92	0.171	≥75	0.23	0.04–1.19	0.080
< 224	1			< 75	1		
**Blood loss** (g)				**BMI** (kg/m^2^)			
≥200	2.70	1.04–7.01	0.041	≥25	2.07	0.76–5.08	0.153
< 200	1			< 25	1		
				**PLH** (mm)			
				≥12.4	4.19	1.57–11.3	0.004
				< 12.4	1		
				**P-A length** (mm) ≥45 < 45	4.061	1.05–15.7	0.042

*BMI* body mass index, *PLH* the maximum vertical length between the upper border of pancreas and the root of left gastric artery, *P-A* pancreas–aorta

### The association between drain amylase level and the PLH and P-A length

We also investigated the association between D-Amy levels between PODs 1 and 3 and PLH because several articles reported D-Amy levels may be used as an indicator of POPF after gastrectomy [3, 6–10] (Table 4). The median D-Amy levels on PODs 1 and 3 were significantly higher in patients with a PLH of ≥12.4 mm compared with those with a PLH of < 12.4 mm (*p* = 0.002, 0.004, respectively) in LG. D-Amy levels in patients with a P-A length ≥ 45 mm was significantly higher only on POD 3 compared with patients with PA-length < 45 mm. However, there were no differences in the D-Amy levels by PLH or P-A length on POD 1 and 3 in OG patients.

**Table 4:** Relationships between the drain amylase level in the drained fluid and PLH or P-A length in patients who underwent laparoscopic and open gastrectomy. Numerical values are presented as the median (range)

		P-A length ≥ 45 ***n*** = 14	P-A length < 45 ***n*** = 101	***p***
**Laparoscopic Gastrectomy**	D-Amy (IU/L) POD 1	596.0 (40–10,484)	419.0 (38–12,154)	0.300
	D-Amy (IU/L) POD 3	323.0 (31–2552)	134.0 (24–5670)	0.044
		**P-A length ≥ 45** ***n*** **= 18**	**P-A length ≥ 45** ***n*** **= 100**	***p***
**Open** **Gastrectomy**	D-Amy (IU/L) POD 1	237.0 (57–14,848)	369.0 (43–16,764)	0.585
	D-Amy (IU/L) POD 3	82.5 (41–1056)	65.0 (12–2855)	0.284
		**PLH ≥ 12.4** ***n*** **= 47**	**PLH < 12.4** ***n*** **= 68**	***p***
**Laparoscopic Gastrectomy**	D-Amy (IU/L) POD 1	657.5 (38–12,154)	319.0 (40–3867)	0.002
	D-Amy (IU/L) POD 3	182.5 (40–5670)	129.0 (24–890)	0.004
		**PLH ≥ 12.4** ***n*** **= 44**	**PLH < 12.4** ***n*** **= 74**	***p***
**Open** **Gastrectomy**	D-Amy (IU/L) POD 1	357.5 (43–14,848)	313.5 (57–16,764)	0.585
	D-Amy (IU/L) POD 3	87.5 (19–2855)	106.0 (12–1594)	0.856

*PLH* the maximum vertical length between the upper border of pancreas and the root of left gastric artery, *P-A* pancreas–aorta, *D-Amy* amylase level in the drained fluid, *POD* postoperative day

## Discussion

In the present study, the relationship between the anatomic location of the pancreas and the incidence of POPF was evaluated on a CT image with a sagittal view in the patients who underwent LG or OG. We found that PLH and P-A length were significantly longer in patients with POPF compared with those without POPF in patients who underwent LG. In the multivariate analysis, PLH ≥12.4 mm and P-A length ≥ 45 mm were extracted as an independent predictor for POPF after LG. The D-Amy levels on PODs 1 and 3 after LG were also significantly higher in patients with a PLH of ≥12.4 mm compared with those with a PLH of < 12.4 mm. These findings indicate that PLH and the P-A length can be a predictor of POPF after LG. However, in patients who underwent OG, the length of PLH and P-A length were not correlated with the frequency of POPF or D-Amy levels on PODs 1 and 3.

The incidence of POPF after LG is reported to be 1.7 to 7.2% [4, 7, 11, 25, 26], and it was reported to be higher compared with after OG [7, 13–15]. POPF is thought to be caused by several factors, including direct damage to the pancreas or lateral thermal injuries by surgical instruments. In addition, blunt pancreatic injury during suprapancreatic lymph node dissection was recently shown to be a cause of POPF in LG [27]. Depending on the anatomic location of the pancreas, compression or retraction of the pancreas by an assistant’s forceps may be required to provide a good surgical view. LG tends to cause excessive pancreas compression because of the restricted instrument axis and lack of delicate tactile sensations. Ida et al. showed pancreatic juice leaking after pancreatic compression using fluorescence imaging with a chymotrypsin probe in a swine laparoscopic gastrectomy model. This suggests that pancreatic compression using the assistant’s forceps can contribute to POPF [28].

Although the measurement method differed in each study, the anatomic position of the pancreas that was evaluated using a preoperative CT image is a predictor of POPF occurrence in LG patients [17, 18]. In the current study, we measured PLH and PLD focusing on the distance between the root of the left gastric artery and the level of the pancreatic body surface. In addition, we investigated the P-A length, a predictor of POPF, reported by Kumagai et al. [18]. Our results showed that long PLH and P-A length were independent predictive factors for POPF in LG, and the odds ratio of PLH (4.19) was higher compared with the P-A length (4.06). It has been well documented that for safe and effective suprapancreatic dissection, it is important to keep the “outermost layer,” meaning the outside of the autonomic sheath around the artery [29]. The anatomical landmark to approach outermost layer is the junction of common hepatic, splenic, and left gastric artery, and the root of the left gastric artery is most useful for recognizing the junction of these arteries on CT images. On the other hand, the nerves sheaths and ganglia surrounding the celiac artery are not usually divided to expose the root of celiac artery located anterior to the aorta in radical gastrectomy. In other words, the depth of the root of celiac artery is not precisely the same as the depth of the outermost layer. Therefore, in theory, the PLH, defined by the distance between the root of the left gastric artery and pancreas, may more accurately represent the depth of the suprapancreatic dissection than the P-A length, defined by the distance between the root of the celiac artery and pancreas. Because CT is a modality that is routinely used for pretreatment diagnosis of gastric cancer in clinical practice, we can easily evaluate the risk of POPF before surgery by measuring PLH in the single slice in the sagittal position.

Another highlight of this study is that it is the first to reveal one of the reasons of the higher incidence of POPF in LG than in OG in the real world. Several authors have indicated the potential risk of pancreatic trauma owing to anatomical location of the pancreas in LG. On the other hand, no previous reports have investigated the relationship between anatomical location and POPF in OG. In the current study, we examined both LG and OG cases during the same period at the same institution, and our results showed the anatomical location of the pancreas in open surgery was not a risk factor for POPF. OG involves less limitation on forceps mobility, and gentle compression or retraction of the pancreas using the human hand could avoid excessive pancreatic parenchymal damage. Therefore, our results indicate that the risk factors for POPF after LG may differ from those after OG. To the best of our knowledge, this is the first report that clearly showed that the anatomic location of the pancreas may be a specific risk factor for POPF after LG.

Several preventive measures for patients who are at high risk for POPF in LG, such as those with a long PLH, were reported to be useful in improving the surgical view, such as additional ports, increasing the pneumoperitoneum pressure, and extreme rotation of the operating table. The best way to avoid pancreas injury is to avoid touching it during the procedure. Tsujiura et al. reported a significant decrease in the incidence of POPF when there was no direct compression of the pancreas during suprapancreatic lymph node dissection in LG patients [27]. Another measure to prevent POPF may be to use a surgical robot, which was developed to overcome several disadvantages that were identified in conventional laparoscopic surgery. Surgical robots provide surgeons with more degrees of freedom through their articulating surgical instruments. Suda et al. reported a single institutional retrospective cohort study, which demonstrated that the incidence of POPF after robotic gastrectomy (RG) was significantly lower compared with that in LG patients (0% vs. 4.3%, *p* = 0.029) [30]. In addition, a multi-institutional prospective study from Japan showed that RG significantly reduced the morbidity rate from 6.4% after LG to 2.45% after RG (*p* = 0.0018), and the incidence of all-grade POPF was only 5.8% [33]. Although further trials are needed, RG may, therefore, reduce the damage and injury to the pancreas and prevent POPF compared with LG and OG.

There were some limitations to the present study. First, this study was a nonrandomized retrospective study. Since some parts of the patient’s background between OG and LG were significantly different, in particular, the OG group contained significantly more cases of advanced stage and those who had undergone TG or D2 dissection, which are risk factors for POPF, than the LG group. Therefore, propensity score matching (PSM) analysis should be considered to eliminate these selection bias, but it is not appropriate to perform PSM owing to the small sample size in this study. Examining the correlation between the anatomic pancreatic position and POPF in advanced gastric cancer patients who undergo LG is required as a next step. Second, diagnostic modalities for POPF may be a concern. The diagnostic criteria for POPF have not been uniformly defined in gastric cancer surgery. Therefore, the definition of POPF after gastrectomy varied with each previous study. The ISGPF criteria were applied in the present study, because these criteria were considered to be more objective compared with other criteria, and they have been used extensively in the field of pancreatic surgery. Although the incidence of POPF in this study was high (24.9%) compared with our daily clinical experience. The incidence of grade B or higher POPF with clinically relevant changes was 4.4%, which was equivalent to that in previous reports [7, 13–15]. However, it is controversial whether patients with BL on ISGPF can be considered as those with POPF, because BL is deemed to be a potential POPF with no clinical impact. In this study, we included BL as one of the diagnostic criteria of POPF owing to the small number of events of grade B or higher POPF. Although several reports have shown that high D-Amy levels are closely correlated with POPF after gastrectomy, further studies with large patient population are required to investigate the relationship between PLH and “true POPF” of grade B or higher.

## Conclusion

In conclusion, PLH may be a specific predictive factor for POPF in patients undergoing LG with suprapancreatic lymph node dissection. Patients who are at a high risk of developing POPF preoperatively can be identified by evaluating the anatomical relationship between the pancreas and the root of the left gastric artery. In patients with a long PLH in particular, careful manipulation around the pancreas is required to prevent POPF in LG.

## Data Availability

The clinical datasets supporting the results of this article are available from the corresponding author.

## Abbreviations

POPF: Postoperative pancreatic fistula
LG: Laparoscopic gastrectomy
OG: Open gastrectomy
PLH: Maximum vertical length between the upper border of the pancreas and the root of the left gastric artery
PLD: Maximum horizontal length between the upper border of pancreas and the root of left gastric artery
P-A length: Maximum length of the vertical line between the pancreatic body surface and the aorta
OR: Odds ratio
CI: Confidence intervals
D-Amy: Amylase level in the drained fluid
BMI: Body mass index
NCD: National clinical database
CT: Computed tomography
POD: Postoperative day
ISGPF: International study group of postoperative pancreatic fistula
PSM: Propensity score matching
